# Data on elemental composition of the medicinal plant *Hymenaea martiana* Hayne (Jatobá)

**DOI:** 10.1016/j.dib.2018.05.142

**Published:** 2018-05-31

**Authors:** Layza Sá Rocha, Daniel Araújo Gonçalves, Daniela Granja Arakaki, Paula Fabiana Saldanha Tschinkel, Nayara Vieira de Lima, Lincoln Carlos Silva de Oliveira, Rita de Cássia Avellaneda Guimarães, Valter Aragão do Nascimento

**Affiliations:** a Group of Spectroscopy and Bioinformatics Applied Biodiversity and Health (GEBABS), Federal University of Mato Grosso do Sul, 549, Campo Grande 790709-00, MS, Brazil; b Federal Universityof Mato Grosso do Sul, Instituto de Química. Federal University of Mato Grosso do Sul, Federal University of Mato Grosso do Sul, 546, 79070900 Campo Grande, MS, Brazil

**Keywords:** Heavy metals, *Elemental compositions of Hymenaea martiana* Hayne, Medicinal plant monitoring

## Abstract

This data article provides elemental compositions profile, determined by inductively coupled plasma-optical emission spectrometry (ICP OES), of the medicinal plant *Hymeneaea martiana* Hayne which belongs of the family Fabaceae (Leguminosae).It is a tree that demonstrates medicinal purposes as antioxidant, immunomodulatory (Boniface et al., 2017) [1], microbial, antiviral, hepatoprotective, gastroprotective (Almeida et al., 2012) [2] and antifungal (Souza et al., 2010) [3].The content of 13 elements (Al, Ca, Cr, Cu, Fe, K, Mg, Mn, Na, Ni, P, S and Zn) in the barks, leaves, tea leaves and bark tea were detected. Data on the cataloging of the plant can be found in the deposit number 64779 of Federal University of Mato Grosso do Sul herbarium, available in www.splink.org.br/form?lang=pt&collectioncode=CGMS.

**Specifications Table**

**Table:** 

**Subject área**	**Chemistry**
**More specific subject area**	*Chemical Analytic*
**Type of data**	*Table*
**How data was acquired**	Microwave digestion System (*Speedwave® four,* Berghof) and ICP OES (Thermo Fisher Scientific, Bremen, Germany, iCAP 6000 Duo)
**Data format**	*Raw, barks, leaves, bark tea and tea leaves of Hymenaea martiana* Hayne (Jatobá).
**Experimental factors**	Pretreatment of samples: (i) The leaves and barks underwent a process of drying and grinding, (ii) Leaves and barks were weighed into digestion vessels using the following mixtures: 2 ml of HNO3 (65% Merck), 1 ml of H2O2 (35%, Merck) and 1 ml of deionized water; (iii) Subsequently, the samples were digested using microwave digestion equipment.
**Experimental features**	Concentration measurements of all samples were analyzed using standard solutions. The detection and quantification limit were obtained. Measuring the elemental compositions (Al, Ca, Cr, Cu, Fe, K, Mg, Mn, Na, Ni, P, S and Zn) in barks, leaves,tea leaves and barks tea of the medicinal plant *Hymenaea martiana* Hayne (Jatobá)
**Data source location**	Campo Grande- Mato Grosso do Sul, Brazil, (Coordinates: 20°31׳11.9"S 54°35׳21.5"W), identified by Dr. Flávio Macedo Alves.
**Data accessibility**	Data included in this article are deposited in the herbarium of Federal University of Mato Grosso do Sul under the number CGMS 64779 available *in:*www.splink.org.br/form?lang=pt&collectioncode=CGMS

**Value of the data**

• The general detection by ICP OES revealed the different levels of macro- and microelements in leaves and barks, tea leaves, and barks tea of the *Hymenaea martiana* Hayne (Jatobá).
• Medicinal plants have therapeutic properties and are nutritionally important because of their mineral contents.
• Heavy metals can accumulate in human body over a long period and may cause adverse effects on human health; therefore, it is important to have a look on good quality control of medicinal plants in order to protect consumers from contamination.
• The data on quantification of mineral concentration in the species *Hymenaea martiana* Hayne obtained can serve as a tool to decide the dosage of preparations from this plant used for medicinal purposes.

## 1 Data

*Hymenaea martiana* Hayne is popularly known in Brazil as ‘‘Jatobá’’. According to popular knowledge, the bark and the leaves of the Jatobá has antibacterial, antispasmodic, antifungal, anti-inflammatory, antioxidant, decongestant, diuretic, expectorant, fortifying, hepatoprotective and vermifuge action. Jatoba tea leaves are used for prostate diseases. On the other hand, jatobáis a tree that has been linked to some relevant pharmacological properties such as antioxidant, immunomodulatory [1], microbial, antiviral, hepatoprotective, gastroprotective [2] and antifungal [3].

The elemental composition data of mineral elements in leaves, leaves tea, bark and bark tea of Jatobá collected in Campo Grande, MS, Brazil, are summarized in Table 1. Some elements were not detected in all samples. Table 1 shows that potassium (K) (with the exception of the bark) is the quantified element with the highest concentration in the leaves, bark tea and tea leaves. Meanwhile, calcium (Ca) is the element of greatest quantification in the bark. Although in the bark the Ca is the element of greater concentration, the potassium follows as the second element of higher content.

**Table 1:** Determination of the elements Al, Ca, Fe, K, Mg, Mn, Na, Ni, P, S and Zn in mg/100 g and Cr and Cu in μg/100 g in fresh leaf, leaf tea, bark, tea bark and standard deviation relative by ICP-OES.

	**Jatobá – Fresh leaf**	**Jatobá – Leaf tea**	**Jatobá – Bark**	**Jatobá - Tea bark**
**Elements**	**mg/100 g**	**mg/100 g**	**mg/100 g**	**mg/100 g**
**Al**	31.56 ± 0.57	< LOD	26.40 ± 0. 68	< LOD
**Ca**	559.54 ± 8.00	19.72 ± 0.99	886.88 ± 4.55	25.59 ± 0.82
**Fe**	37.59 ± 0.24	< LOD	37.0 ± 0.37	0.19 ± 0.01
**K**	688.58 ± 8.1	280.21 ± 14.88	460.22 ± 6.13	199.49± 6.89
**Mg**	151.98 ± 2.31	20.93 ± 0.50	26.95 ± 0.57	11.18 ± 0.29
**Mn**	109.32 ± 1.18	3.60 ± 0.05	33.36 ± 0.38	3.03 ± 0.03
**Na**	21.06 ± 0.21	15.17 ±0.51	20.98 ± 1.07	13.46 ± 0.42
**Ni**	0.17 ± 0.01	0.092 ± 0.01	0.13 ± 0.05	0.054 ± 0.01
**P**	179.37 ± 0.83	34.30 ± 0.62	79.45 ± 0.28	20.26 ± 0.18
**S**	194.14 ± 2.78	26.07 ± 0.20	120.76 ± 0.40	58.70 ± 0.28
**Zn**	1.85 ± 0.04	0.79 ± 0.01	1.42 ± 0,02	0.83 ± 0.08
**Elements**	**µg/100 g**	**µg/100 g**	**µg/100 g**	**µg/100 g**
**Cr**	380 ± 10	100 ± 10	230 ± 20	188 ± 10
**Cu**	140 ± 20	< LOD	< LOD	< LOD

< LOD - Analyte concentrations were below the limits of detection.

According to Table 1, nickel (Ni) is the quantified element of lower concentration in the bark, bark tea and tea leaves. Copper (Cu) was detected only in the leaves and was the element of lower quantification. Although in the leaves Cu is the element of lower concentration, Ni follows as the second element to have lower determined content. Thus, Ni remains the lowest element in the samples. Table 1 shows that aluminium was detected only in the leaves and bark of Jatobá. Aluminium, copper and iron were not detected in tea leaves. In the bark tea the aluminium and the copper were not detected and in bark only the copper. The leaves were the only sample in which all elements were determined.

## 2 Experimental design, materials and methods

### 2.1 Collection and identification of vegetable material

The leaves and barks of *H. martiana* Hayne were collected in October 2017 in an urban area of the city of Campo Grande, Mato Grosso do Sul (Coordinates: 20 ° 31׳11.9 "S 54 ° 35׳21.5" W). All samples collected (*H. martiana Hayne* - Fabaceae) were deposited on 11/03/2017 at the herbarium of the Federal University of Mato Grosso do Sul and identified by the biologist Dr. Flávio Macedo Alves, deposit number 64779 CGMS - 64779 (*H. martiana* Hayne- Fabaceae).

### 2.2 Sample preparation and digestion

The leaves and bark were set to dry in an oven at 50 °C for 48 h until the stabilization of their weights. The dried samples were crushed for spraying, separately, with a portable stainless steel electric grinder to obtain a very fine powder. A quantity (0.250 g) of each, powder of the leaves and bark were weighed on an analytical balance and placed separately in flasks (in triplicate).

In the flasks containing the previously weighed samples were added 2 ml of nitric acid (HNO_3_ 65% Merck), 1 ml of hydrogen peroxide (H_2_O_2_ 35%, Merck) and 1 ml of deionized water (Millipore, Milli-Q Biocel Water Purification System, Germany). For the blank solutions were added 2 ml of HNO_3_, 1 ml of H_2_O_2_ and 1 ml of deionized water in others flasks. Subsequently, the samples were placed on digestion vessels of microwave digestion system (BERGHOF Products + Instruments GmbH - Speedwave 4 - Microwave Digestion System), using the operating program specified in Table 2.

**Table 2:** Operating conditions for the microwave digestion system.

**Step**	**Power (%**^a^**)**	**Temperature (°C)**	**Ramp time (min)**	**Hold Time (min)**	**Pressure (Bar)**
1	50	150	10	5	30
2	80	190	5	15	35
3	0	50	1	10	25

a 100 % power correspond to 1450 W.

After process of digestion by microwave system, the contents of the vessels were transferred to the 50 ml Falcons tubes, and then filled to 30 ml with deionized water and was directed to the ICP OES reading.

### 2.3 Preparation of leaves and bark tea

To prepare the leaf and bark tea, it was added in 2 beakers of 250 ml the equivalent to 100 ml of deionized water, separately, and each one was directed to be heated in a thermal blanket. For the verification of the temperature variation, a thermometer was placed together and when the temperature reached 100 °C, 4.00 g of fresh leaf previously weighed were added in beaker 1 and 4.00 g of pre-weighed fresh bark were added in beaker 2. It cooked for 15 min where the temperature was maintained and monitored. After the time finished the blanket was turned off and the beaker was capped for 10 min for a better infusion and then withdrawn. After cooling, separately, each teas were filtered 3 times to remove impurities generating a final volume of 30 ml to the Falcon tube and used for reading on ICP OES.

### 2.4 Elemental analysis by ICP OES technique

After the process of digestion the concentrations of the elements were determined by the technique of Inductively Coupled Plasma Optical Emission Spectrometry - ICP OES (Thermo Fisher Scientific, Bremen, Germany, iCAP 6000 Duo). The selected emission lines (wavelength in nm) for the determination of elements in bark, leaves, teas and the operating conditions of ICP OES are summarized in Table 3.

**Table 3:** Instrumental analytical conditions for ICP OES of element analyses.

**Parameters**	**Setting**
RF Power (W)	1250
Sample flow rate (L min^−1^)	0.35
Plasma gas flow rate (L min^−1^)	12
Integration time (s)	5
Stabilization time (s)	20
Nebulization time (psi)	30
Plasm view	Axial
Gas (99.999%)	Ar
Analytes	Emission line (nm)
	Al 396.100; Ca 422.673;
	Co 228.616; Cr 267.716;
	Cu 324.754; Fe 259.940;
	K 766.490; Mg 279.553;
	Mn 257.610; Na 588.995;
	Ni 221.647; P 214.914;
	S 180.701; Zn 213.856;

### 2.5 Calibration curves

Basic stock solutions containing 1000 mg L^**−**1^ (SpecSol, Quimlab, Brazil) of each elements Al, Ca, Cr, Cu, Fe, K, Mg, Mn, Na, Ni, P, S and Zn were used to prepare a diluted standard solution. For each element detected (Table 4), a Limit of Quantification (LOQ), Limit of Detection (LOD) and correlation coefficient (*R*^2^) were established. An external calibration curve (Linear Equation) was established for all studied elements. The calibration curve of the blank was used to determine the LOD and LOQ of the calibration method.

**Table 4:** Parameters of calibration obtained external calibration, correlation coefficient (*R*^2^), limit of detection (LOD) and limit of quantification (LOQ) by using ICP OES.

	**Linear Equation**	***R***^**2**^	**LOD (mg L**^**−1**^**)**	**LOQ (mg L**^**−1**^**)**
Al	*Y* = 11,885.95C + 611.15	0.9995	0.03	0.1
Ca	*Y* = 40,820.51C + 3455.69	0.9991	0.02	0.08
Cr	*Y* = 4553.11C + 26.77	0.9998	0.001	0.004
Cu	*Y* = 7706.92C + 371.69	0.9997	0.002	0.006
Fe	*Y* = 5783.58C + 81.22	0.9999	0.02	0.07
K	*Y* = 12,786.02C + 178.39	0.9999	0.01	0.04
Mg	*Y* = 29,851.11C + 718.86	0.9994	0.01	0.03
Mn	*Y* = 31,761.44C + 24.41	0.9990	0.001	0.004
Na	*Y* = 133,026.16C + 6064.82	0.9992	0.0001	0.0005
Ni	*Y* = 2876.32C + 5.11	0.9996	0.002	0.008
P	*Y* = 113.41C + 0.98	0.9977	0.01	0.04
S	*Y* = 7.47C + 1.80	0.9987	0.0005	0.002
Zn	*Y* = 5764.08C + 18.70	0.9963	0.0002	0.0005

The limit of detection (LOD) represents the minimum measured content from which it is possible to determine the presence of a particular analyte, without, however, having sufficient statistical accuracy to be able to quantify accurately. It corresponds to the minimum concentration that can be distinguished from blank [4].

When the analytical method involves the use of linear calibration, the limit of detection is calculated by the standard deviation of the blank on the calibration curve. The limit of detection can be calculated according to Eq. (1) [4]:

(1) LOD=3×s/S

where, *s* and *S* are the standard deviation of the response and the angular coefficient of the calibration graph (instrument sensitivity).

The quantification limit (LOQ) is defined as the lowest analyte concentration that can be quantified in the sample, with acceptable accuracy and precision. When the method involves the use of a linear calibration, the limit of quantification is calculated as follows [4]:

(2) LOQ=10×s/S

After the calibration curves made with the mono elementary solutions of Al, Ca, Cr, Cu, Fe, K, Mg, Mn, Na, Ni, P, S and Zn the read of the samples obtained with the microwave assisted digestion process and the teas development procedure, was separately made. The concentrations of the different elements in these samples were determined using the corresponding standard calibration curves obtained using standard solutions of the elements of interest. Triplicate analyses were performed for each sample. The results were expressed as the mean of the triplicates ± standard deviation. The program used was Microsoft Office Excel 2007.
